# Physical working conditions over time: a repeated cross-sectional study in German employees

**DOI:** 10.1186/s12995-024-00423-8

**Published:** 2024-06-10

**Authors:** Johannes Beller, Julia Graßhoff, Batoul Safieddine

**Affiliations:** Hannover Medical School, Center for Public Health and Health Care, Medical Sociology Unit, Carl- Neuberg-Str. 1, 30625 Hannover, Germany

**Keywords:** Occupational Health, Working conditions, Trends, Inequality

## Abstract

**Background:**

This study aimed to examine time trends in physical working conditions across and within occupational groups in Germany between 2006 and 2018.

**Methods:**

Logistic regression analyses were conducted using data from the BIBB/BAuA Employment Surveys in 2006, 2012, and 2018, with a total sample size of 59,006 participants. The study investigated changes in various self-reported occupational exposure measures over time, along with demographic shifts in the workforce.

**Results:**

The results showed overall improvements in most occupational exposure measures during the study period, alongside an aging and upskilling workforce. However, exposure to awkward postures, microorganisms, and, to a lesser extent, noise increased. Substantial variation was observed between occupational groups, with more favourable trends among white-collar high-skilled and blue-collar low-skilled workers, and less favourable trends among white-collar low-skilled and blue-collar high-skilled workers.

**Conclusions:**

While trends in physical working conditions in Germany are partly promising, some exposures are worsening, and substantial inequalities between occupations persist. As occupational exposures remain common, there is still a need for targeted interventions to improve working conditions, particularly in higher-risk occupations.

**Supplementary Information:**

The online version contains supplementary material available at 10.1186/s12995-024-00423-8.

## Background

Physical working conditions refer to the physical aspects of the work environment that affect the health and well-being of workers. These factors include ergonomics, such as heavy lifting, as well as exposure to environmental hazards, such as fumes [1]. The impact of physical working conditions on employees’ health and well-being is well-established [2]. Numerous studies have demonstrated the link between poor physical working conditions and the development of physical health problems, mental health problems and reduced work ability [3–7]. For example, heavy lifting and awkward postures have been associated with musculoskeletal disorders [8, 9]. Furthermore, exposure to hazardous substances, such as fumes, has been linked to respiratory diseases and cancers [10, 11]. Lastly, stressful physical work environments, characterized by high noise levels, poor lighting, and extreme climate, have been associated with increased levels of mental health conditions [12, 13].

Given the importance of physical working conditions for workers’ health, it seems crucial to study time trends in these risk factors [2]. However, studies examining this topic are lacking. Among the few existing studies, Havet and Penot [14] explored trends in exposures to physical working conditions in France using data from the Medical Monitoring Survey of Professional Risks conducted in 2003, 2010, and 2017. The authors found that while exposure to awkward postures increased between 2003 and 2017, exposure to hazardous physical environments decreased sharply. However, these trends varied across worker subgroups, with blue-collar workers experiencing increased inequalities in exposure to certain physical demands. In another study, Gustavsson and colleagues [15] investigated trends in occupational exposure to chemicals in Sweden between 1980 and 2013 via a job exposure matrix. They found that the exposure generally declined over time, however, again with marked social inequalities between social strata. As a last example, Kauppinen et al. [16] also investigated trends in occupational exposure to chemicals in Finland via a job exposure matrix. They also found that exposures generally declined over time, however, with substantial differences between exposure agents. In summary, while some studies have investigated time trends in physical working conditions, the evidence remains limited and fragmented. Given the significant health implications of these exposures, more research is needed to understand the evolution of physical working conditions across different contexts and worker populations.

Changes over time in working conditions might be expected given that many countries experienced significant demographic, economic, technological, and regulatory shifts in recent years. For example, Germany, as many other industrialized countries, has experienced a notable aging of its workforce [17]. Simultaneously, women’s labor force participation rates have been rising [18]. Economically, Germany saw a continued shift towards a service-based economy, with the tertiary sector’s share of total employment increasing steadily. Certain occupational sectors such as healthcare and education, which often involve different physical working conditions compared to traditional industries, experienced especially pronounced growth [19]. Technological advancements, particularly in the context of automation and digitalization, also gained momentum in recent decades [20]. Finally, several important regulatory changes related to occupational health and safety were implemented in recent years. For example, Germany further implemented the EU Directive 2009/104/EC on minimum safety and health requirements for the use of work equipment by workers in 2002 and 2015, thus enhancing safety standards especially related to physical work.

In light of these contextual factors, the current study aims to further investigate time trends in physical working conditions. It goes beyond most previous studies by examining these trends across different occupational groups, including white-collar high-skilled, white-collar low-skilled, blue-collar high-skilled, and blue-collar low-skilled occupations, and by using self-reported exposures instead of job exposure matrices. Thus, the current study clarifies (a) how physical working conditions have evolved over time in Germany and (b) whether these trends differ between white-collar and blue-collar occupations of varying skill levels. We ask: “How have physical working conditions changed in Germany?”.

## Methods

### Sample

Data were collected from the BIBB/BAuA Employment Surveys conducted in 2006, 2012, and 2018. These cross-sectional studies gather information about the German workforce and have been carried out periodically, with the most recent survey taking place in 2018 [21–23]. Participants were required to be at least 15 years old, work a minimum of 10 h per week, and possess sufficient German language proficiency to be eligible for the survey.

The surveys employed a random-digital-dialing method for both landline and mobile numbers in each survey. The interviews, which had an average duration of approximately 40 min, covered topics such as sociodemographic variables, work activities, working conditions, and health. Informed consent was obtained from all participants, and the procedures were carried out in compliance with German law and the 1964 Helsinki declaration and its subsequent amendments. The BAuA ethics committee granted approval for the surveys, with the most recent approval being EK007_2017 on January 9, 2017. After removing participants with missing values through listwise deletion (*n* = 1042, representing about 2% of the original sample size), the final sample consisted of *N* = 59,006 participants (N_2006_ = 19,787, N_2012_ = 19,609, N_2018_ = 19,610).

### Measures

The physical working conditions were assessed using a questionnaire that asked participants about the frequency of exposure to various conditions during their work activities: “Let us now come to various working conditions and whether this occurs frequently, sometimes, rarely or never during your activity as […]” (translated from the original German version). Thus, these measures reflect workers’ subjective perceptions of their physical working conditions. The physical working conditions included were: (1) Standing (“Work standing up. How often does this happen?“); (2) Heavy Lifting (“Loads of more than 20 kg for male participants, and 10 kg for female participants”); (3) Fumes (“Work with smoke, dust or under gases, vapours”); (4) Extreme Climate (“Work under cold, heat, moisture, humidity or draughts”; (5) Dirt (“Working with oil, grease, dirt”). (6) Awkward Postures (“Working in a bent, squatting, kneeling position or working overhead”); (7) Poor Light (“Work in bright light or with poor or insufficient illumination”). (8) Noise (“Working under noise”). (9) Microorganisms (“Handling microorganisms such as pathogens, bacteria, moulds or viruses”). Participants could respond to each condition with “frequently”, “sometimes”, “rarely”, or “never”. For the analyses in the current study, the responses were coded dichotomously as “1” for “frequently” and “0” for all other response options (however, similar results are obtained when using other operationalizations; Table A5, Figure A1).

The covariates were operationalized as follows. Age: Age was operationalized in years. Gender: Gender was operationalized as male or female. Working hours were operationalized as the average weekly working hours reported by participants. Occupation: Participants were classified into four occupational groups based on the International Standard Classification of Occupations (ISCO): White-Collar High-Skilled (ISCO: 1 Managers, 2 Professionals, 3 Technicians), White-Collar Low-Skilled (ISCO: 4 Clerical Support Workers, 5 Service Workers), Blue-Collar High-Skilled (ISCO: 6 Skilled Agricultural workers, 7 Craft workers), and Blue-Collar Low-Skilled (ISCO: 8 Machine Operators, 9 Elementary Occupations).

### Data Analysis

First, descriptive statistics of all variables are reported. Then, to determine trends, logistic regression analyses are conducted predicting working conditions via age (in years), gender (0 = male; 1 = female) and time period (scaled as a metric variable between 0 and 1 with 2006 = 0, 2012 = 0.5, and 2018 = 1, such that the effect of time period can be interpreted as the change between 2006 and 2018; however, similar results are obtained when comparing the 2006 and 2018 wave directly). For each working condition a separate regression analysis was conducted. Trends were also analyzed in stratified samples according to occupational group. All regression analyses are weighted according to the design weights provided by the surveys and were controlled for age and gender (ethnicity was not available and thus could not be controlled for; similar results are obtained when using just weights without covariates, as seen in Figure A2).

## Results

As depicted in Table 1, participants were on average 44.85 years old (SD = 11.13) and worked 38.53 h per week (SD = 12.20), with 50.3% being female. The most prevalent physical working conditions were standing (48.6%) and exposure to noise (20.8%), while the least common were exposure to light (8.2%) and fumes (9.9%). Comparing occupational groups, blue-collar workers in high-skilled occupations (BC-HS) had the highest prevalence of awkward postures (35.5%), standing (84.8%), heavy lifting (41.9%), exposure to fumes (32.1%), climate (39.3%), dirt (47.2%), and noise (50.0%). In contrast, white-collar workers in high-skilled occupations (WC-HS) had the lowest prevalence of most physical working conditions, except for exposure to microorganisms (11.9%). Blue-collar workers in low-skilled occupations (BC-LS) had the second-highest prevalence of most physical working conditions, while white-collar workers in low-skilled occupations (WC-LS) generally fell between the WC-HS and BC-LS groups.

**Table 1:** Physical working conditions and sociodemographic characteristics across occupational groups

	Stratified by Occupational Group					
	Overall	WC-HS	WC-LS	BC-HS	BC-LS	
N	59,006	33,871	11,719	7224	6192	
Postures (%)	13.0	8.0	9.9	35.5	19.5	
Standing (%)	48.6	36.9	49.9	84.8	68.2	
Lifting (%)	18.8	11.3	19.1	41.9	32.1	
Fumes (%)	9.9	3.7	8.3	32.1	21.5	
Climate (%)	16.2	6.6	17.3	39.3	39.7	
Dirt (%)	12.9	4.4	8.6	47.2	27.9	
Light (%)	8.2	5.6	8.3	15.4	14.0	
Noise (%)	20.8	14.0	13.2	50.0	37.7	
Microorganisms (%)	10.2	11.9	10.0	5.1	6.9	
Age (mean (SD))	44.85 (11.13)	45.54 (10.95)	43.70 (11.39)	43.14 (11.15)	45.23 (11.17)	
Female (%)	50.3	52.6	73.8	15.4	33.9	
Working Hours (mean (SD))	38.53 (12.20)	39.67 (11.81)	33.68 (12.29)	41.92 (9.98)	37.55 (13.72)	
Occupational Group (%)						
WC-HS	57.4	100.0	0.0	0.0	0.0	
WC-LS	19.9	0.0	100.0	0.0	0.0	
BC-HS	12.2	0.0	0.0	100.0	0.0	
BC-LS	10.5	0.0	0.0	0.0	100.0	

### Time trends

As depicted in Table 2, physical working conditions changed on a descriptive level over time. Comparing 2006 to 2018, the prevalence of standing decreased from 51.6 to 45.4%, while lifting and carrying heavy loads also decreased from 20.1 to 16.8%. Exposure to fumes, poor climatic conditions, dirt, and poor lighting conditions all decreased by 3.2%, 3.5%, 2.9%, and 1.0%, respectively. In contrast, the prevalence of exposure to microorganisms increased from 8.1 to 12.6%. The prevalence of awkward postures and exposure to noise remained relatively stable. The changes in physical working conditions from 2006 to 2018 varied across occupational groups (Table A1, Table A2, Table A3, Table A4). White-collar occupational groups generally experienced increases in awkward postures, noise, and exposure to microorganisms, while blue-collar groups were subject to more varied changes across physical working conditions.

**Table 2:** *Physical Working Conditions and Sociodemographic Characteristics Across Time*

	Stratified by Time Period				
	Overall	2006	2012	2018	
N	59,006	19,787	19,609	19,610	
Postures (%)	13.0	12.2	14.2	12.4	
Standing (%)	48.6	51.6	48.8	45.4	
Lifting (%)	18.8	20.1	19.4	16.8	
Fumes (%)	9.9	11.8	9.4	8.6	
Climate (%)	16.2	18.0	16.2	14.5	
Dirt (%)	12.9	14.7	12.3	11.8	
Light (%)	8.2	8.7	8.3	7.7	
Noise (%)	20.8	21.2	20.2	20.8	
Microorganisms (%)	10.2	8.1	9.9	12.6	
Age (mean (SD))	44.85 (11.13)	41.30 (10.46)	46.06 (10.70)	47.22 (11.31)	
Female (%)	50.3	48.7	52.5	49.8	
Working Hours (mean (SD))	38.53 (12.20)	38.83 (12.94)	38.52 (12.05)	38.23 (11.56)	
Occupational Group (%)					
WC-HS	57.4	53.1	55.1	64.0	
WC-LS	19.9	21.2	21.4	17.0	
BC-HS	12.2	14.2	12.7	9.8	
BC-LS	10.5	11.4	10.8	9.2	

**Fig. 1:**
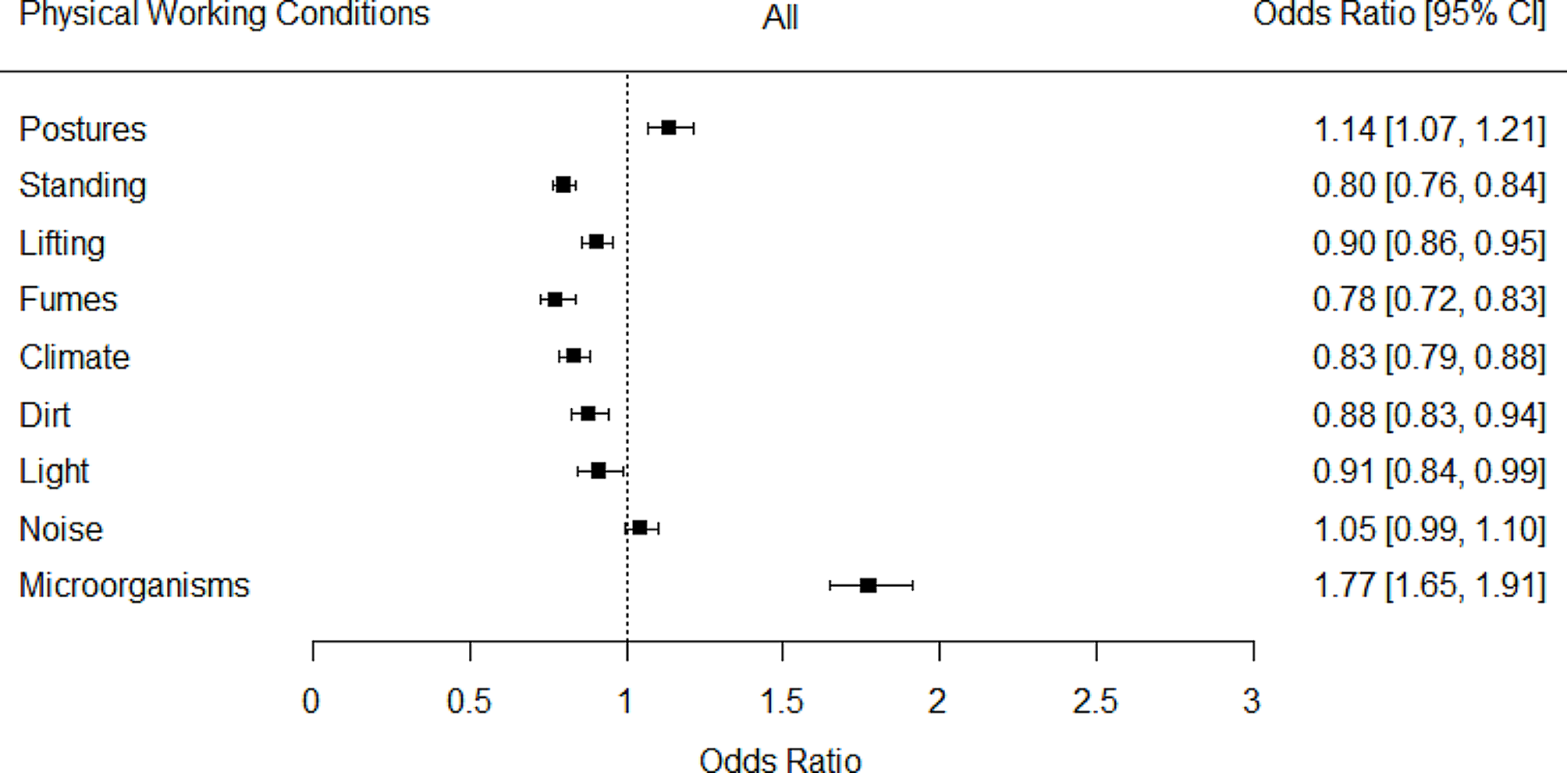
Trends in physical working conditions

Next, as depicted in Fig. 1, regression analyses were used to study time trends in physical working conditions. The odds of exposure to awkward postures (OR = 1.14, 95%-CI: [1.07, 1.21]) and microorganisms (OR = 1.77, 95%-CI: [1.65, 1.91]) increased significantly over time in the whole sample. Conversely, the odds of exposure to standing (OR = 0.80, 95%-CI: [0.76, 0.84]), lifting (OR = 0.90, 95%-CI: [0.86, 0.95]), fumes (OR = 0.78, 95%-CI: [0.72, 0.83]), adverse climate (OR = 0.83, 95%-CI: [0.79, 0.88]), dirt (OR = 0.88, 95%-CI: [0.83, 0.94]), and lighting (OR = 0.91, 95%-CI: [0.84, 0.99]) decreased. Furthermore, a small but non-significant increase in noise (OR = 1.05, 95%-CI: [0.99, 1.10]) was observed.

**Fig. 2:**
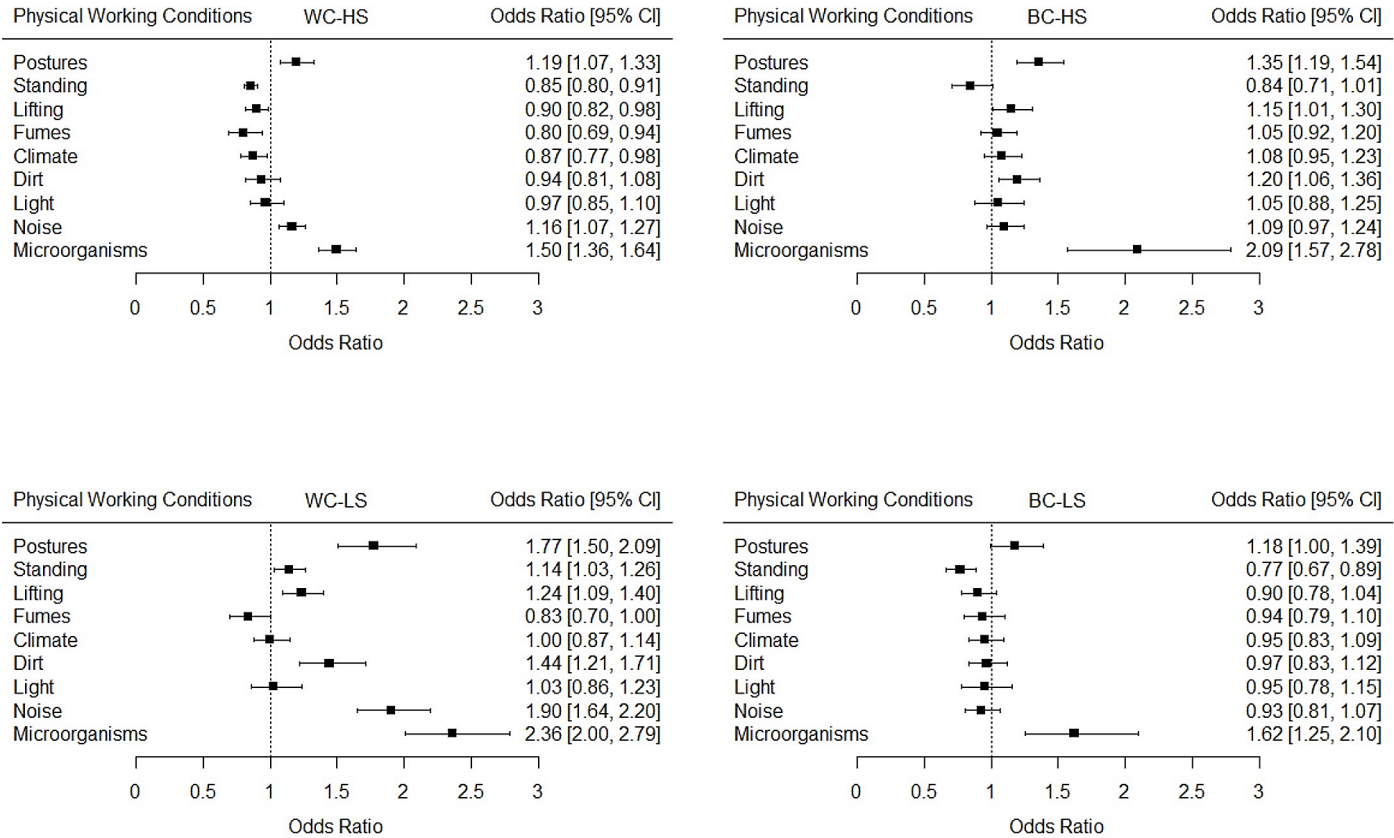
Trends in physical working conditions in occupational groups. *Notes.* WC-HS = White-Collar High-Skilled Occupational Group; WC-LS = White-Collar Low-Skilled Occupational Group; BC-HS = Blue-Collar High-Skilled Occupational Group; BC-LS = Blue-Collar Low-Skilled Occupational Group

Next, as depicted in Fig. 2, stratified regression analyses were used to study stratified trends according to occupational groups. The results showed diverging trends in physical working conditions between the occupational groups. For the BC-HS and WC-LS groups, the odds of many physical working conditions increased significantly over time or were stable. In contrast, the WC-HS and BC-LS groups generally experienced decreasing or stable trends in exposure to most physical working aspects, except for increases in awkward postures, microorganisms and, for WC-HS, exposure to noise. Similar trends were observed in several robustness analyses (see Table A5, Figures A1 and A2).

## Discussion

We examined trends in physical working conditions across and within occupational groups in Germany between 2006 and 2018. Overall improvements in most self-reported occupational exposure measures were found in the whole sample during the study period, coinciding with an aging and upskilling workforce. However, some exposures, such as awkward postures, exposure to microorganisms, and, to a lesser extent, exposure to noise, increased over time on average. Notably, the trends varied substantially between occupational groups: White-collar high-skilled and blue-collar low-skilled workers experienced more favourable trends, while white-collar low-skilled and blue-collar high-skilled workers faced less favourable trends.

These results both confirm and contradict previous studies. Some previous studies have reported similar findings of improvements in certain occupational exposures over time [14–16]. For example, Havet et al. (2022) found that exposure to hazardous physical environments decreased sharply in France between 2003 and 2017. However, they also observed an increase in the prevalence of exposure to severe physical constraints. Similarly, our study found that while overall trends were encouraging, some exposures like awkward postures worsened. Going beyond most previous studies, we distinguished between skill levels within the comparatively broad categories of blue-collar and white-collar workers. This approach revealed important novel differences. Blue-collar high-skilled and white-collar low-skilled workers faced particularly unfavourable trends, especially regarding awkward postures, exposure to microorganisms, and noise. Meanwhile, white-collar high-skilled workers and blue-collar low-skilled workers generally experienced more positive or stable trends. The only negative trends for these groups were significant increases in awkward postures, microorganisms, and noise. These findings suggest that occupational skill level plays a key role in shaping temporal trends in physical working conditions [24–26].

### Explanations

There are several possible explanations for the trends observed in physical working conditions across occupational groups in Germany between 2006 and 2018. One potential factor is the ongoing process of structural change of work. This involves tertiarization, a shift from industrial to service-based economies, as well as the trend towards a knowledge society, characterized by an increasing emphasis on education and skills. Both structural changes imply a reduction in certain physical exposures associated with traditional industrial work, as also observed in this study [27]. Additionally, service sector occupations often involve more sedentary work, sustained awkward postures (e.g., in office or computer-based jobs), and potential exposure to microorganisms (e.g., in healthcare or customer-facing roles). The rise in service sector employment may therefore also partially explain the increases we observed in these specific physical working conditions [28]. Another key driver of change might be the digitalization of work activities. The adoption of digital technologies and automation may reduce physical demands in most occupations but increase the need for sustained postures, as also found in this study [29]. Even within the industrial and manufacturing sectors, the nature of work activities and associated physical demands may have changed due to technological advancements [20]. Lastly, occupational safety measures and regulations could also contribute to improvements in working conditions. Stricter safety standards, increased awareness, and targeted interventions may have helped reduce exposure to certain extreme physical hazards, particularly in high-risk blue-collar high-skilled occupations. In line with this hypothesis, most consistent improvements were also found among those working in blue-collar low-skilled occupations in this study. However, future studies are needed to elucidate the concrete mechanisms for the observed changes in physical working conditions over time.

Additionally, the high proportion of women in the white-collar low skilled group, which reported the most adverse changes over time, raises important questions about the role of gender in determining the perceptions of physical strain in the workplace over time. Research suggests that gender norms contribute to disparities in how men and women perceive and report physical risks [30, 31]. For example, women have been reported to be more likely to report physical symptoms and express concerns about workplace hazards compared to men [32–35]. Moreover, the concentration of women in certain occupations, such as healthcare and social work, may expose them to unique physical challenges and risks over time. These gender-related factors could contribute to the observed trends in physical working conditions in our study and should be investigated further by future studies.

### Implications

The findings contribute to the understanding of the potential impact of the changing world of work on workers’ health on a population level [36]. The results suggest that musculoskeletal disorders related to severe physical working conditions such as exposure to heavy lifting might decline over time on average. However, substantial variation in trends were also observed across occupational groups such that more research on the health implications within specific occupations is needed.

At the same time, the study results also have implications for explaining the negative health trends observed in younger and middle-aged adults, in line with the expansion of morbidity hypothesis [37–42]. Perhaps the decreased levels of physical activity at work and the shift towards more sedentary occupations might have contributed to increased cardiometabolic and functional health problems. This hypothesis is also in line with recent increasing trends in type 2 diabetes [43]. For example, recent evidence points towards a significant increase in type 2 diabetes complications and comorbidities in working-age individuals [44, 45]. However, again, more research is needed to formally study the reasons for the observed changes in health over time [28, 46, 47].

Another critical factor to consider when interpreting the observed trends in physical working conditions is the aging of the workforce. As previously noted, the average age of the study sample increased substantially from 41.3 years in 2006 to 47.2 years in 2018, mirroring the broader demographic shift in Germany’s working population. This aging trend could have profound implications for workers’ physical health and well-being. Older workers may be more susceptible to the detrimental effects of physical strain, as age-related changes in musculoskeletal function, recovery capacity, and overall resilience can increase their vulnerability to work-related health complaints [48, 49]. For example, the healing process following an injury may be slower and less complete in older individuals, leading to prolonged disability and increased risk of recurrent and chronic health conditions [50]. This might result in higher health risks due to poor working conditions over time, even with regards to the physical working conditions with more stagnating trends.

From a practical perspective, the results of this study emphasize the need for targeted interventions to improve working conditions, particularly in the emerging higher risk occupations such as WC-LS and BC-HS groups [47]. Prevention strategies should focus on reducing exposure to and handling of harmful physical working conditions, such as awkward postures, microorganisms, and noise. Despite the observed improvements in the majority of physical working conditions, prevalence remains high, thus underscoring the need for continued efforts to improve working conditions across all occupational groups.

### Limitations

The current study has several limitations that should be considered when interpreting the results. First, the data relied on self-reported measures of physical working conditions, which reflect workers’ subjective perceptions rather than objective measurements. These perceptions may be subject to recall bias and social desirability bias [51]. It must be emphasized that subjective perceptions can differ significantly from objective assessments, and future studies should aim to additionally investigate how objective measures of physical working conditions have changed over time. In a similar vein, the study utilized single-item measures to operationalize various physical working conditions, which may not fully capture the complexity, frequency and multidimensional nature of these exposures [1]. Multi-item scales or objective measurements could provide more comprehensive assessments of working conditions. Furthermore, while the study considered several socio-demographic factors, there may be unmeasured stratification variables, such as company size, sector and region, that could also be important in determining the observed trends [15]. Future studies should also investigate the degree to which socio-demographic changes explain the observed trends in working conditions. Finally, the study was conducted in Germany, and the findings may not be generalizable to other countries. As such, multi-national studies are needed.

## Conclusion

Despite some limitations, we were able to comprehensively analyse trends in self-reported physical working conditions in Germany. We found overall improvements in most physical working conditions over time, with the notable exceptions of exposure to awkward postures, microorganisms, and noise. Additionally, substantial variation was observed between occupational groups, with less favourable trends among white-collar low-skilled and blue-collar high-skilled workers. Future studies should replicate and expand upon these results by using more robust exposure assessment methods, by considering additional stratification variables and by further exploring the mechanisms behind the observed trends. Doing so might help unravel the reasons for the recently observed rise of morbidity in working-age adults [52].

### Electronic supplementary material

Below is the link to the electronic supplementary material.

Supplementary material 1

## Data Availability

The data that support the findings of this study are available from the Federal Institute for Vocational Education and Training (BIBB) at https://www.bibb.de/en/2815.php.
