# Supplementary material for: Physical working conditions over time: a repeated cross-sectional study in German employees
Source: J Occup Med Toxicol. 2024 Jun 10;19:24. doi: 10.1186/s12995-024-00423-8 (PMC11165766; doi:10.1186/s12995-024-00423-8)
Supplement: Supplementary file 1 — Supplementary material 1 [file 12995_2024_423_MOESM1_ESM.docx]

**Appendix**

Table A1. *Physical Working Conditions and Sociodemographic Characteristics Across Time in the White-Collar High-Skilled Occupational Group*

|  | **Stratified by Time Period** | | | | |
| --- | --- | --- | --- | --- | --- |
|  | **Overall** | **2006** | **2012** | **2018** |  |
| N | 33871 | 10514 | 10803 | 12554 |  |
| Postures (%) | 8.0 | 7.1 | 9.1 | 7.9 |  |
| Standing (%) | 36.9 | 38.4 | 36.7 | 35.7 |  |
| Lifting (%) | 11.3 | 12.0 | 11.9 | 10.2 |  |
| Fumes (%) | 3.7 | 4.3 | 3.3 | 3.4 |  |
| Climate (%) | 6.6 | 7.4 | 6.4 | 6.2 |  |
| Dirt (%) | 4.4 | 5.0 | 3.9 | 4.3 |  |
| Light (%) | 5.6 | 5.9 | 5.5 | 5.5 |  |
| Noise (%) | 14.0 | 13.3 | 13.2 | 15.4 |  |
| Microorganisms (%) | 11.9 | 9.9 | 11.9 | 13.7 |  |
| Age (mean (SD)) | 45.54 (10.95) | 42.14 (10.29) | 46.54 (10.53) | 47.52 (11.18) |  |
| Female (%) | 52.6 | 50.7 | 55.1 | 52.0 |  |
| Working Hours (mean (SD)) | 39.67 (11.81) | 40.46 (12.54) | 39.81 (11.69) | 38.87 (11.21) |  |

Table A2. *Physical Working Conditions and Sociodemographic Characteristics Across Time in the White-Collar Low-Skilled Occupational Group*

|  | **Stratified by Time Period** | | | | |
| --- | --- | --- | --- | --- | --- |
|  | **Overall** | **2006** | **2012** | **2018** |  |
| N | 11719 | 4197 | 4192 | 3330 |  |
| Postures (%) | 9.9 | 7.9 | 10.6 | 11.4 |  |
| Standing (%) | 49.9 | 50.7 | 49.4 | 49.6 |  |
| Lifting (%) | 19.1 | 17.8 | 19.8 | 19.8 |  |
| Fumes (%) | 8.3 | 9.6 | 6.9 | 8.2 |  |
| Climate (%) | 17.3 | 18.0 | 16.3 | 17.7 |  |
| Dirt (%) | 8.6 | 8.4 | 8.1 | 9.5 |  |
| Light (%) | 8.3 | 8.3 | 8.3 | 8.0 |  |
| Noise (%) | 13.2 | 11.2 | 12.7 | 16.4 |  |
| Microorganisms (%) | 10.0 | 7.6 | 9.4 | 13.8 |  |
| Age (mean (SD)) | 43.70 (11.39) | 39.74 (10.74) | 45.14 (11.01) | 46.89 (11.23) |  |
| Female (%) | 73.8 | 73.5 | 75.1 | 72.4 |  |
| Working Hours (mean (SD)) | 33.68 (12.29) | 33.43 (13.05) | 33.76 (11.91) | 33.89 (11.76) |  |

Table A3. *Physical Working Conditions and Sociodemographic Characteristics Across Time in the Blue-Collar High-Skilled Occupational Group*

|  | **Stratified by Time Period** | | | | |
| --- | --- | --- | --- | --- | --- |
|  | **Overall** | **2006** | **2012** | **2018** |  |
| N | 7224 | 2812 | 2491 | 1921 |  |
| Postures (%) | 35.5 | 33.4 | 36.6 | 37.2 |  |
| Standing (%) | 84.8 | 86.5 | 83.5 | 84.1 |  |
| Lifting (%) | 41.9 | 42.5 | 40.9 | 42.5 |  |
| Fumes (%) | 32.1 | 33.4 | 30.8 | 31.9 |  |
| Climate (%) | 39.3 | 40.1 | 38.5 | 39.4 |  |
| Dirt (%) | 47.2 | 47.9 | 44.6 | 49.7 |  |
| Light (%) | 15.4 | 15.4 | 15.1 | 15.8 |  |
| Noise (%) | 50.0 | 51.4 | 48.9 | 49.5 |  |
| Microorganisms (%) | 5.1 | 3.9 | 5.1 | 6.8 |  |
| Age (mean (SD)) | 43.14 (11.15) | 40.06 (10.27) | 44.97 (10.80) | 45.28 (11.81) |  |
| Female (%) | 15.4 | 14.2 | 16.9 | 15.4 |  |
| Working Hours (mean (SD)) | 41.92 (9.98) | 42.08 (10.20) | 41.72 (9.93) | 41.93 (9.70) |  |

Table A4. *Physical Working Conditions and Sociodemographic Characteristics Across Time in the Blue-Collar Low-Skilled Occupational Group*

|  | **Stratified by Time Period** | | | | |
| --- | --- | --- | --- | --- | --- |
|  | **Overall** | **2006** | **2012** | **2018** |  |
| N | 6192 | 2264 | 2123 | 1805 |  |
| Postures (%) | 19.5 | 18.0 | 21.2 | 19.3 |  |
| Standing (%) | 68.2 | 71.3 | 68.6 | 63.9 |  |
| Lifting (%) | 32.1 | 34.3 | 31.3 | 30.2 |  |
| Fumes (%) | 21.5 | 23.6 | 19.6 | 21.0 |  |
| Climate (%) | 39.7 | 39.9 | 39.4 | 39.8 |  |
| Dirt (%) | 27.9 | 30.0 | 26.0 | 27.6 |  |
| Light (%) | 14.0 | 13.9 | 14.6 | 13.6 |  |
| Noise (%) | 37.7 | 39.2 | 36.9 | 36.7 |  |
| Microorganisms (%) | 6.9 | 5.9 | 6.2 | 9.0 |  |
| Age (mean (SD)) | 45.23 (11.17) | 41.83 (10.45) | 46.71 (10.64) | 47.75 (11.58) |  |
| Female (%) | 33.9 | 35.9 | 36.0 | 28.8 |  |
| Working Hours (mean (SD)) | 37.55 (13.72) | 37.25 (14.37) | 37.63 (13.56) | 37.83 (13.06) |  |

Table A5. *Physical Working Conditions and Sociodemographic Characteristics Across Time With Full Answer Options*

|  | **Stratified by Time Period** | | | | |
| --- | --- | --- | --- | --- | --- |
|  | **2006** | **2012** | **2018** |  |  |
| n | 19787 | 19609 | 19610 |  |  |
| Postures (%) |  |  |  |  |  |
| Never | 61.3 | 58.4 | 59.6 |  |  |
| Rarely | 14.2 | 14.8 | 15.1 |  |  |
| Sometimes | 12.3 | 12.6 | 12.9 |  |  |
| Often | 12.2 | 14.2 | 12.4 |  |  |
| Standing (%) |  |  |  |  |  |
| Never | 15.3 | 15.3 | 14.7 |  |  |
| Rarely | 19.5 | 20.7 | 21.0 |  |  |
| Sometimes | 13.6 | 15.1 | 18.9 |  |  |
| Often | 51.6 | 48.8 | 45.4 |  |  |
| Lifting (%) |  |  |  |  |  |
| Never | 42.3 | 40.6 | 48.8 |  |  |
| Rarely | 23.2 | 25.2 | 22.0 |  |  |
| Sometimes | 14.4 | 14.8 | 12.3 |  |  |
| Often | 20.1 | 19.4 | 16.8 |  |  |
| Fumes (%) |  |  |  |  |  |
| Never | 66.8 | 69.3 | 72.9 |  |  |
| Rarely | 12.4 | 12.5 | 10.6 |  |  |
| Sometimes | 9.0 | 8.8 | 7.9 |  |  |
| Often | 11.8 | 9.4 | 8.6 |  |  |
| Climate (%) |  |  |  |  |  |
| Never | 55.3 | 54.2 | 58.3 |  |  |
| Rarely | 12.8 | 14.5 | 12.7 |  |  |
| Sometimes | 13.8 | 15.1 | 14.5 |  |  |
| Often | 18.0 | 16.2 | 14.5 |  |  |
| Dirt (%) |  |  |  |  |  |
| Never | 65.4 | 66.5 | 69.8 |  |  |
| Rarely | 10.8 | 11.2 | 9.9 |  |  |
| Sometimes | 9.1 | 10.0 | 8.6 |  |  |
| Often | 14.7 | 12.3 | 11.8 |  |  |
| Light (%) |  |  |  |  |  |
| Never | 66.0 | 65.0 | 64.3 |  |  |
| Rarely | 13.6 | 14.4 | 15.5 |  |  |
| Sometimes | 11.7 | 12.3 | 12.5 |  |  |
| Often | 8.7 | 8.3 | 7.7 |  |  |
| Noise (%) |  |  |  |  |  |
| Never | 51.9 | 50.1 | 46.5 |  |  |
| Rarely | 12.4 | 14.2 | 16.0 |  |  |
| Sometimes | 14.5 | 15.5 | 16.7 |  |  |
| Often | 21.2 | 20.2 | 20.8 |  |  |
| Microorganisms (%) |  |  |  |  |  |
| Never | 80.2 | 75.6 | 70.1 |  |  |
| Rarely | 6.0 | 7.0 | 8.5 |  |  |
| Sometimes | 5.7 | 7.5 | 8.8 |  |  |
| Often | 8.1 | 9.9 | 12.6 |  |  |
| Age (mean (SD)) | 41.30 (10.46) | 46.06 (10.70) | 47.22 (11.31) |  |  |
| Female (%) | 48.7 | 52.5 | 49.8 |  |  |
| Working Hours (mean (SD)) | 38.83 (12.94) | 38.52 (12.05) | 38.23 (11.56) |  |  |
| Occupational Group (%) |  |  |  |  |  |
| WC-HS | 53.1 | 55.1 | 64.0 |  |  |
| WC-LS | 21.2 | 21.4 | 17.0 |  |  |
| BC-HS | 14.2 | 12.7 | 9.8 |  |  |
| BC-LS | 11.4 | 10.8 | 9.2 |  |  |

Figure A1. *Trends in “Never Being Exposed” to Physical Working Conditions*


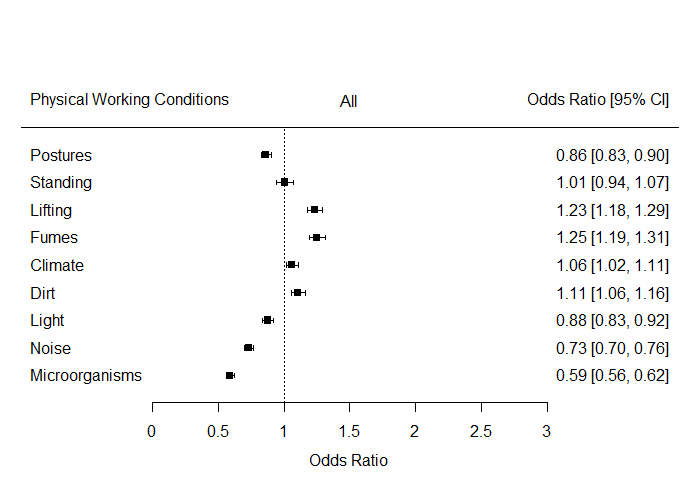


Figure A2. *Trends in Physical Working Conditions Without Covariates*


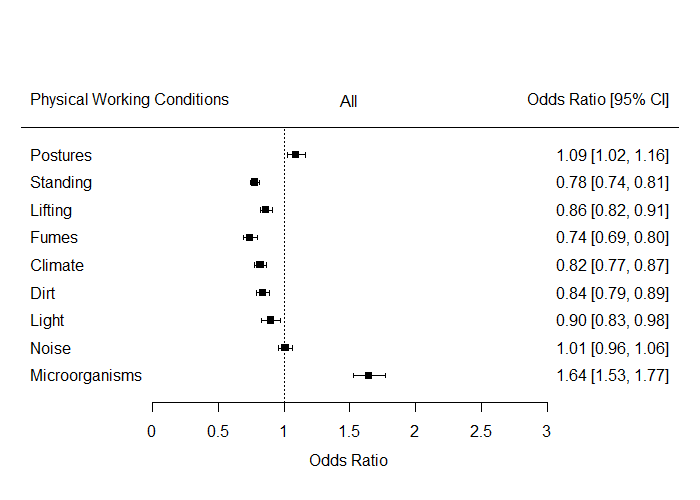


*Notes.* In this analysis physical working conditions were only predicted by time period, without controlling for age and gender.
